# Neuropsychological Evaluation and Quantitative EEG in Patients with Frontotemporal Dementia, Alzheimer’s Disease, and Mild Cognitive Impairment

**DOI:** 10.3390/brainsci13060930

**Published:** 2023-06-08

**Authors:** Letteria Tomasello, Leonardo Carlucci, Angelina Laganà, Santi Galletta, Chiara Valeria Marinelli, Massimo Raffaele, Pierluigi Zoccolotti

**Affiliations:** 1 Department of Clinical and Experimental Medicine, University of Messina, 98122 Messina, Italy; ltomasello@unime.it (L.T.); massimo.raffaele@unime.it (M.R.); 2 Faculty of Medicine and Dentistry, Sapienza University of Rome, 00185 Rome, Italy; 3 Learning Sciences Hub, Department of Humanities, Letters, Cultural Heritage and Educational Studies, Foggia University, 71121 Foggia, Italy; leonardo.carlucci@unifg.it; 4 Department of Biomedical and Dental Sciences, Morphological and Functional Images, 98122 Messina, Italy; angelina.lagana@unime.it; 5 Réseau Hospitalier Neuchâtelois (RHNe), Service de Neurologie et Neuroréadaptation, 2000 Neuchâtel, Switzerland; santi.galletta@rhne.ch; 6 Tuscany Rehabilitation Clinic, 52025 Montevarchi, Italy; pierluigi.zoccolotti@crtspa.it; 7 Department of Psychology, Sapienza University of Rome, 00185 Rome, Italy

**Keywords:** dementia, Alzheimer’s, MCI, EEG, neuropsychological profile

## Abstract

This study analyzed the efficacy of EEG resting state and neuropsychological performances in discriminating patients with different forms of dementia, or mild cognitive impairment (MCI), compared with control subjects. Forty-four patients with dementia (nineteen patients with AD, and seven with FTD), eighteen with MCI, and nineteen healthy subjects, matched for age and gender, underwent an extensive neuropsychological test battery and an EEG resting state recording. Results showed greater theta activation in posterior areas in the Alzheimer’s disease (AD) and Fronto-Temporal Dementia (FTD) groups compared with the MCI and control groups. AD patients also showed more delta band activity in the temporal-occipital areas than controls and MCI patients. By contrast, the alpha and beta bands did not discriminate among groups. A hierarchical clustering analysis based on neuropsychological and EEG data yielded a three-factor solution. The clusters differed for several neuropsychological measures, as well as for beta and theta bands. Neuropsychological tests were most sensitive in capturing an initial cognitive decline, while increased theta activity was uniquely associated with a substantial worsening of the clinical picture, representing a negative prognostic factor. In line with the Research Domains Framework (RDoC) perspective, the joint use of cognitive and neurophysiological data may provide converging evidence to document the evolution of cognitive skills in at-risk individuals.

## 1. Introduction

Dementia is a clinical syndrome caused by neurodegeneration and characterized by a progressive deterioration in cognitive ability and capacity for independent living [1,2,3]. It comprises a group of neurodegenerative disorders related to cognitive decline that influence memory, language, social abilities, and executive functions [4,5]. With the progression of cognitive decline, patients with dementia show difficulties in performing daily life activities and may become entirely dependent on caregivers [6]. Indeed, family caregivers support patients with dementia, and many caregivers experience substantial psychological and economic burdens [7,8]. 

Patients with dementia are heavy consumers of health services. This leads to direct costs for public health care services, given that no current treatment is highly effective, but it can only mitigate cognitive impairments [9,10]. Moreover, with the disease progression, particularly in the later stages, the patient’s brain shows neuroinflammation and irreversible synaptic losses [11,12]. Thus, the treatment of dementia implies many difficulties in economic and therapeutic terms. For this reason, early diagnosis and detection of neuronal damage allow for both a timely therapeutic intervention to manage the symptoms and an adequate preparation of patients and caregivers. Yet, the diagnosis of dementia is still a debated question. Thus, along with the establishment of clinical standards [4,5], diagnostic biological biomarkers are developed (e.g., machine learning algorithms for neuroimaging data; Ref. [13]).

Neurophysiological analysis with electroencephalography (EEG) is a non-invasive tool that allows the dynamic analysis of electrical brain activity. EEG signals are reliable for distinguishing patients with dementia from subjects without dementia (controls) or other neurological disorders [14,15]. Rossini et al. [16] found that the use of quantitative EEG (qEEG) allows the identification of patients with mild cognitive impairment (MCI) from control subjects. Moreover, to distinguish patients with Alzheimer’s Disease (AD) or MCI from control subjects, recent reviews indicated that diffused slowing of the EEG is a specific diagnostic marker: patients with AD showed increased power in lower frequency bands (delta and theta) and a decrease in alpha and beta power [17,18,19]. Following these results, Meghdadi et al. [20] investigated whether resting-state EEG measures can be potential biomarkers for detecting and assessing cognitive decline in patients with MCI and AD. Patients in the AD group showed increased delta and theta power and a decreased power in the alpha frequency band. A small but significant increase in the power of slow frequencies, localized to temporal areas, was also found in patients with MCI.

The relationship between the EEG signals and the cognitive abilities of older subjects can provide information about cognitive alterations [21]. High resting-state theta power in older subjects was linked with more pronounced cognitive impairment [22]. There were also positive correlations between resting theta power and cognitive deficits in healthy subjects and those with MCI [23,24]. Some studies investigated the EEG signals and progression from MCI to AD and found that patients who progressed to AD showed more delta, theta, and lower alpha and beta power than those who did not [25,26,27]. Other studies indicated that these EEG patterns (higher theta power and less beta power) were associated with subsequent cognitive decline measured via neuropsychological tests [28,29]. A study by Finnigan and Robertson [22] on healthy older subjects examined the degree to which resting EEG band power was associated with cognitive performance. Theta power was significantly correlated with immediate and delayed verbal recall, attention, and executive function measures, whereas delta and alpha did not correlate with any cognitive measures. This study suggested that high resting theta power in healthy older subjects was associated with better cognitive function. This finding is in line with the link between theta power and cognition but not with other literature data indicating that theta power can point to subsequent cognitive decline and MCI or dementia [22].

Hence, although the EEG has become a suitable, accurate, and sensitive biomarker for the identification of neuronal and cognitive dynamics in different types of dementia, such as AD, MCI, or Fronto-Temporal Dementia (FTD) [8], the nature of the relationship between EEG signals and cognitive abilities in dementia is still unclear. Thus, it is not presently clear whether the increased power of slow frequency bands in patients with dementia is a marker of overall cognition or whether it is related to specific cognitive domains or the type of dementia.

There were two main aims of this study: first, to highlight the EEG patterns, expressed as percentage changes of delta, theta, alpha, and beta bands in different cortical areas, and to examine whether these patterns can be significantly different, allowing to discriminate between patients with AD, FTD, and MCI, compared with control subjects. 

**Figure 1:**
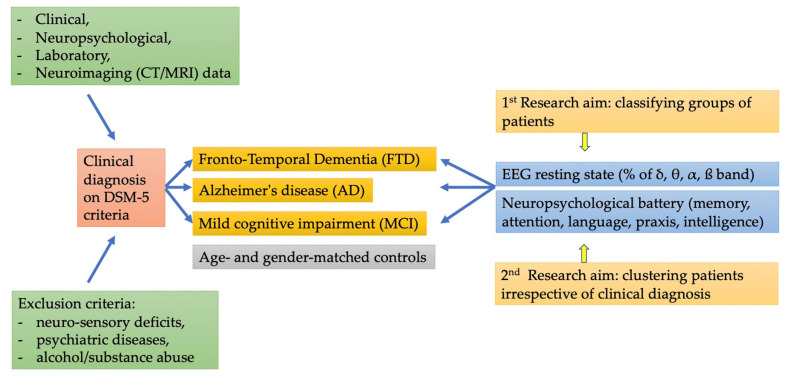
Block diagram of the study.

A second aim was to jointly examine EEG and neuropsychological functions from a transdiagnostic perspective using a clustering approach [30]. While classical diagnoses maintain a clinical utility, the recent NIH Research Domains Framework (RDoC [31]) proposes to examine links across domains (e.g., neurophysiological, and cognitive) by considering individual variations, including multiple case groups over and above diagnostic categories. A block diagram of the research aims is in Figure 1.

## 2. Materials and Methods

### 2.1. Participants

Forty-four patients attending the Alzheimer’s Evaluation Unit (a Neurological Clinic sited in Messina) participated in the study: 19 patients with AD (mean age in years = 69.8; SD = 0.48); 7 patients with FTD (mean age in years = 68.8; SD = 0.58), and 18 patients with MCI (mean age in years = 67; SD = 0.45). There were no differences among groups for age (F_(3,60)_ = 1.3; n.s.), years of schooling (F_(3,60)_ = 0.1; n.s.), and gender (X^2^ = 3.4, n.s.) (see Table 1). The diagnosis of dementia syndromes was based on clinical, neuropsychological, laboratory, and neuroimaging data according to current research criteria for AD, FTD, and MCI. Patients with severe neuro-sensory deficits, psychiatric diseases, and alcohol/substance abuse were excluded. 

The diagnosis of dementia syndromes was based on clinical, neuropsychological, laboratory, and neuroimaging (either CT or MRI) data according to current research criteria for AD, FTD, and MCI based on the DSM-5 criteria [5]. For initial diagnostic evaluation, all participants routinely underwent a neurological examination and a detailed anamnesis and neuropsychological assessment. Patients with severe neuro-sensory deficits, psychiatric diseases, and alcohol/substance abuse were excluded. Disease duration was measured as time since first noticing the symptoms, as reported by caregivers and was also based on clinical records. For differential diagnosis between suspected AD and MCI, only mild disease stages were considered with a Mini-Mental State Examination (MMSE) score of at least 20 [32,33]. 

In the case of suspected FTD, disease progression was additionally estimated via the Frontal Behavioral Inventory [34]. Following the test manual, scores between 25 and 30 points indicated early and mild stages [35]. When no local atrophy pattern was visible on MRI of the brain at 3.0 Tesla, 18F-fluorodeoxyglucose positron emission tomography (FDG-PET) was additionally applied in the subsample of seven patients with FTD. FDG-PET revealed a typical bifrontal hypometabolism. Particularly, we aimed to eliminate the possibility of underlying AD pathology in patients with suspected FTD [36].

Nineteen healthy subjects matched for age and gender with the patients were enrolled as a healthy control group. These participants had no dementia or other neurological or psychiatric disorders and showed a performance within expected limits at the MMSE (see the next paragraph). They were recruited through online ads. All participants were enrolled after the completion of a screening interview conducted via telephone and a comprehensive in-office clinical and personal information questionnaire to assess their global cognitive functioning. Additionally, in this case, participants with neuro-sensory deficits, psychiatric diseases, and alcohol/substance abuse were excluded from the control group.

All participants provided written informed consent for participating in this study. All methods were performed following the ethical standards of the Declaration of Helsinki.

### 2.2. Neuropsychological Assessment

All patients underwent a neuropsychological test battery to assess the extent of impairment in the cognitive area. We selected a set of neuropsychological tests for analysis to cover important cognitive domains. All tests have an Italian standardization for the critical ages tested. We evaluated overall cognition based on the MMSE. We examined specific cognitive domains through the following tests: Memory: immediate and delayed recall of the Babcock Story Recall Test (BSRT); Verbal Short Term Memory: Serial Repetition Test of Two-syllable Words; Visuospatial Working Memory: Corsi’s Block Tapping Test (CBTT); Attention: Trail-Making Test A and B (TMT); Language: Verbal Fluency Tests; Praxis: Constructional Apraxia Test (CAT); and Fluid Intelligence: Raven’s Colored Progressive Matrices (CPM).

Overall cognition: The MMSE consists of thirty items that assess orientation, short and long-term memory, language, attention, visuospatial skills, and the ability to follow simple verbal and written commands. This easy-to-use and relatively quick neuropsychological test is often employed to assess the overall cognitive status [37]. We referred to norms for the Italian population considering age and education corrections [38].

Memory: The Babcock Story Recall Test (BSRT) [39] measures immediate and delayed recall. The examiner reads a brief story, and the participant must provide immediate recall. Then, the story is repeated, and a delayed recall is obtained after ten minutes. Italian norms are available for this test. The Serial Repetition Test of Two-syllable Words [40] measures verbal short-term memory. In this test, the examiner presents a sequence of unrelated two-syllable words that the subject must repeat immediately after the presentation. The test is preceded by an example. The examiner begins with a two-word sequence; if the subject correctly repeats 2 out of 3 stimuli, the examiner moves to a longer set (one more word). The span is given by the longest sequence for which at least 2 out of 3 stimuli are correctly repeated. Corsi’s Block Tapping Test [40] measures visuospatial working memory. This test consists of nine cubes fastened in random order to a blackboard; each time the examiner taps the blocks in a prearranged sequence, the patient must copy the tapping pattern, which involves a series of blocks of increasing span length to be tapped by the patient in a forward (memory span) or backward (working memory span) manner. Thus, the task is like the verbal digit span but with a visual presentation.

Attention: The Trail Making Test (TMT) [41] measures visual attention and task switching. It consists of 25 circles distributed over a sheet of paper. In part A, the circles are numbered from 1 to 25, and the patient should draw lines to connect the numbers in ascending order. The patient is instructed to connect the circles as quickly as possible without lifting the pen or pencil from the paper. The time to connect the “trail” is measured. If the patient makes an error, the examiner immediately points it out to allow the patient to correct it. Errors affect the patient’s score as the error correction is included in the time completion for the task. The test is interrupted if the patient has not completed both parts within five minutes.

Language: The Verbal Fluency Test is a short test of linguistic functioning [42,43,44]. It consists of two tasks: category or semantic fluency and letter or phonemic fluency. Semantic verbal fluency is measured by the number of words produced within a restricted category. Name categories are semantic colors, animals, and fruits. Concerning phonemic fluency, participants were instructed to generate words beginning with the test letters F, A, and S, spelled out loud by the examiner in this order. Examinees were given 60 s to name as many words as possible, beginning with the first letter; the procedure was then repeated for the two remaining letters. The participants were informed of inadmissible words (repetitions, proper names, or words with different inflections sharing the same root) to be eliminated from the analysis. Application and scoring criteria were derived from Senhorini et al. [45]. Instructions were followed by examples using the letter P to illustrate correct and incorrect words. The participants are given 1 min to produce as many unique words as possible within a semantic category (category fluency) or starting with a given letter (letter fluency). The participant’s score in each task is the number of unique correct words.

Constructional praxis: The Constructional Apraxia Test (CAT) [43,44] was used to measure the ability to consistently copy the elements that constitute the geometric bidimensional models presented by the examiner. The figures derive from those used in the test of Arrigoni and De Renzi [46]. After the run-in, the examiner presents the figures printed on the upper half of the paper; the participant is asked to copy them below as precisely as possible. A score of 4 is given if the reproduction is perfect, 1 if the copy is partially defective, and 0 if the reproduction is unrecognizable or if there is a “closing-in” (despite the instructions, the subject follows the contour of the figure printed above).

Fluid Intelligence: The Raven’s Coloured Progressive Matrices (CPM) [47] is a non-verbal intelligence test representative of general intellectual capacity or the “g” factor proposed by Spearman. The CPM was developed to assess children aged from 5 to 11 years old, mentally disabled individuals as well as older individuals. The items are organized in ascending difficulty throughout three sets (A, Ab, and B). On average, set B is more difficult than set Ab, which is more difficult than set A. The items consist of a drawing with a missing part, which the individual needs to complete by choosing one among six alternative responses. There is only one correct answer for each item. The respondents score one for each correct response and zero for each wrong response. The minimum score is 0, and the maximum score is 36. 

### 2.3. EEG Evaluation

The Micromed^®^ electroencephalograph, and subsequent data processing with the System Plus software, were used. The EEG activity was recorded continuously from 19 sites by using electrodes set in an elastic cup and positioned according to the International System 10–20 Jasper: Fp1, Fp2, F7, F3, Fz, F4, F8, T3, C3, Cz, C4, T4, T5, P3, Pz, P4, T6, O1, and O2. Patients were instructed to sit with closed eyes and relax. A member of staff controlled the subjective and online EEG traces, alerting the patient any time there were signs of EEG drowsiness. EEG signal was recorded at 250 Hz from the participants in a 30 min resting state in which intermittent light stimulation (ILS) and hyperpnea (HP) were also executed. Participants were required to remain awake during the entire recording. Standard 16-channel montage was used according to the 10–20 International System. The raw EEG tracks were analyzed using the short-time Fourier Transform analysis, and each channel was decomposed in delta (0.5–3 Hz), theta (4–7.5 Hz), alpha (8–12.5 Hz), and beta (13–30 Hz) frequency bands. For each channel, the occurrence of the frequency bands was expressed as a percentage. 

In the ILS, a lamp was placed ~30 cm in front of the patient and the patient was asked to fixate the lamp. Stimulation commenced as the patient closed his eyes. Stimulation was delivered at an intensity of 18 Hz, and then following the sequence 2, 6, 8, 10, 15, 20, 30, 40, 50, and 60 Hz. Every stimulation lasted 8 s with 4 s of pause between stimulations. The patient was asked to open his eyes ~4 s after stimulation onset and close them after ~4 s. In the HP protocol, the patients were asked to take deep breaths for 3 to 5 min in room air with a respiratory rate of approximately 20 to 30 breaths per minute, and the EEG recording continued for at least 3 min afterward. These tests were used to prompt epileptiform abnormalities, and if epileptiform abnormalities appeared, the EEG was excluded from the group sample. No patient showed any epileptic signs during the recording.

#### 2.3.1. EEG Scoring

Epochs with ocular, muscular, and other types of artifacts, were discarded by two skilled EEG technicians. The resulting track, with an average duration of 8 min, was subjected to qEEG analysis software. We obtained a quantitative analysis for each patient and each channel; the percentages expressed values of the four frequency spectra analyzed (δ, θ, α, and ß).

#### 2.3.2. Statistical Analyses

Neuropsychological tests: First, we examined performance on neuropsychological tests using one-way ANOVAs. In the case of significant group effects, post hoc comparisons were carried out via the Fisher’s Least Significant Difference (LSD) post hoc test. In the case of MMSE, we used raw scores. For all other neuropsychological tests, we used equivalent scores based on Italian standardized norms [40]; in all cases, scores vary between 0 (lowest) and 4 (highest).

EEG Analysis: For each band (delta, theta, alfa, and beta), we carried out a separate ANOVA with group (4 levels: controls, FTD patients, AD patients, and MCI patients) as a between-subjects factor and EEG channel (19 levels: Fp1, Fp2, F7, F3, Fz, F4, F8, T3, C3, Cz, C4, T4, T5, P3, Pz, P4, T6, O1, and O2) as a repeated measure. Whenever appropriate, main effects and interactions were further examined using the LSD post hoc test.

We performed a post hoc power analysis using the G*power3 [48]. This analysis indicated that this study had adequate statistical power at a large effect size level (F = 0.40; *p* < 0.05; ref. [49]) but low power at a small to moderate effect size (F = 0.10–0.25; *p* < 0.05). The small sample size indicates the need for caution in generalizing the findings of this study to the population with dementia.

Next, we classified cases (patients and controls) based on the performance of neuropsychological tests measured using equivalent scores (e.g., ability to modulate attention, visuomotor coordination ability, recall words both semantically and phonologically, etc…), and the EEG bands. This is in line with the hypothesis that similar patterns of performance may be associated with groups of items that share some commonalities. To this end, neuropsychological scores and EEG bands were entered in multivariate classification methods to test and/or confirm groups of cases able to replicate a priori diagnostic classification based exclusively on a single cognitive pattern (the MMSE).

## 3. Results

### 3.1. Participants

Table 1 shows the socio-demographic data and neuropsychological results for the clinical groups of patients (AD, FTD, and MCI) and the control group. 

For all neuropsychological variables, there was a significant main effect for the group factor (in all cases, at least *p* < 0.001). Post hoc comparisons conducted via the LSD test indicated that patients with AD were impaired in all domains compared to controls. Their performance was also lower than the patients with MCI in all cognitive tests, except short-term memory. Finally, they showed worse performances than patients with FTD in MMSE, long-term memory, and visual-spatial memory. As compared to controls, patients with FTD were impaired in MMSE, short-term memory, attention, semantic and phonemic verbal fluency, and praxis, while they were not different in long-term memory, visual-spatial memory, and fluid intelligence. Patients with MCI were impaired compared to controls in short-term memory and praxis; all other comparisons were insignificant. 

### 3.2. EEG

#### 3.2.1. Theta Bands

The ANOVA on theta bands highlighted the significance of the main effect of the EEG channel (F_(18,1062)_ = 3.29; *p* < 0.0001) indicating different theta activation as a function of the electrode position (with generally lower theta activity in posterior areas). The group factor (F_(3,59)_ = 3.77; *p* < 0.05) indicated generally greater theta activation in FTD (25.4%) and AD (22.0%) than in controls (18.8%) and MCI patients (15.6%). Additionally, the EEG channel x group interaction was significant (F_(54,1062)_ = 2.47; *p* < 0.0001). As shown in Figure 1, except in the frontal area, AD and FTD patients showed greater involvement of theta bands than controls; MCI patients generally showed lower involvement of theta bands than the other patients, as well as (to a smaller degree) than control subjects.

**Figure 2:**
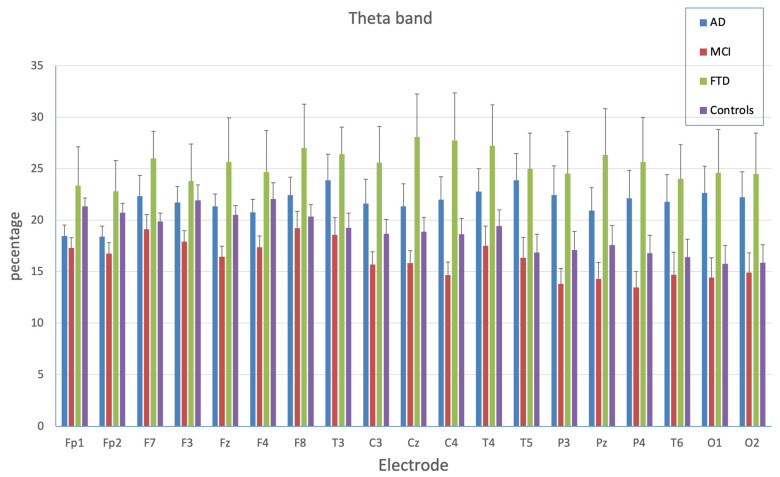
Percentage of theta activation as a function of electrode position and group. Bars indicate standard errors of means (SEM).

As shown in Figure 2, there was similar theta activity for each channel in FTD and AD patients, while lower theta activity was present in the posterior areas in controls and MCI patients. In particular, planned comparisons showed that FTD and MCI patients differed in theta activation for each EEG channel examined (at least *p* < 0.05), with larger theta activation for FTD patients than MCI patients; AD patients showed larger theta activation than MCI patients for electrodes T5, O1, and O2 (at least *p* < 0.05), and a trend was evident for the other posterior areas (in particular for the electrodes P3, P4, T6, and T3). Control subjects showed larger theta activation than MCI patients in the FP1, FP2, F2, and F4 electrodes (at least *p* < 0.05). The AD group showed larger theta activation than controls in posterior areas, with a significant difference in O1, O2, and T5 (at least *p* < 0.05) and a trend approaching significance in T3, P3, P4, and T6. FTD patients showed greater theta activation than controls in all electrodes examined (at least *p* < 0.05), except for more anterior areas, as highlighted by the absence of significant group differences for FP1, FP2, F3, F4, and CZ, and a trend of significance for P3 and T6 recording. FTD and AD patients showed a similar pattern of theta activity, except for FP1, FP2, and CZ (at least *p* < 0.05), which was more pronounced among FTD participants.

#### 3.2.2. Delta Bands

**Figure 3:**
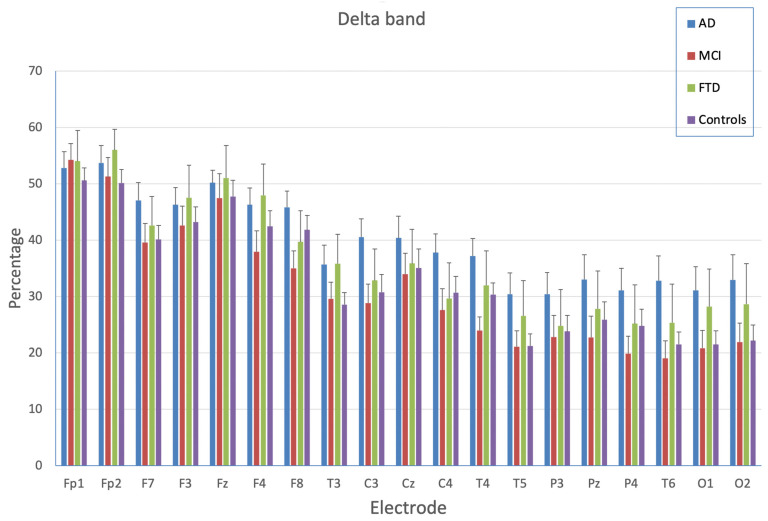
Percentage of delta activation as a function of electrode position and group. Bars indicate standard errors of means (SEM).

The ANOVA on delta bands highlighted the significance of the main effect of the EEG channel (F_(18,1062)_ = 95.96; *p* < 0.0001), with larger delta activity in the frontal area (especially in the pre-frontal area) than in the “central area” bands and especially the parietal-occipital areas. The group’s main effect was insignificant (F_(3,59)_ = 1.60; *p* = 0.12), indicating similar delta activity in the four groups. The EEG channel x group interaction was significant (F_(54,1062)_ = 1.38; *p* < 0.05): as shown in Figure 3, delta activity was greater among AD patients than in the other groups (that were similar among each other). The exploration of the interaction showed that AD patients and control subjects differed in temporal-occipital areas: more activity was observed among AD patients than in controls in C3, T5, T6, O1, and O2 (at least *p* < 0.05) with a similar trend approaching significance in T3 and T4. The AD group showed higher delta activity than MCI patients in F8, C3, C4, T4, T5, P4, T6, O1, and O2 (at least *p* < 0.05) and a trend in the same direction (but only approaching significance) for the F7, F4, and P2 channels. There were no group differences in delta activity for any other channel examined.

#### 3.2.3. Beta Bands

The ANOVA on beta bands did not highlight either the main effect of group (F_(3,59)_ = 1.83, n.s.) or the EEG channel x group interaction (F_(54,1062)_ = 1.10; n.s.), indicating similar beta activity in the four groups, both in general and in every EEG channel examined. The unique significant effect was the main effect of the EEG channel (F_(18,1062)_ = 5.92; *p* < 0.0001), with generally higher activity in the central areas. 

#### 3.2.4. Alpha Bands

The ANOVA on alpha did not highlight either the main effect of group (F_(3,59)_ = 1.77, n.s.) or the EEG channel x group interaction (F_(54,1062)_ = 1.10, n.s.), indicating similar alpha activity in the four groups, both in general and for every EEG channel examined. The unique significant effect was the main effect of the EEG channel (F_(18,1062)_ = 75.42; *p* < 0.0001), with higher activity in the parietal-occipital areas than in the central and especially the frontal ones.

#### 3.2.5. Cluster Analysis

We carried out a cluster analysis of neuropsychological assessment scores and alpha, beta, theta, and delta EEG bands. A hierarchical and non-hierarchical approaches method (a dual process clustering procedure [50,51]) was applied to identify subgroups of cases (patients and controls) with similar cognitive responses profile and EEG patterns.

Different from the previous diagnostic classification, in an explorative manner, we did not a priori specify the number of clusters. Of note, we used z-scores to yield equal metrics and equal weighting. Additionally, no multivariate outliers were detected as assessed using the Mahalanobis Distance test (below the cut-off χ^2^ = 29.53, *p* < 0.001). Out of the total sample, we discharged three cases that completely lacked random data on the neuropsychological and EEG measures. The final database was composed of 63 cases.

We performed a hierarchical clustering analysis using the squared Euclidean distance matrix with the Ward’s linkage method [52] for forming clusters. Since there is no formal stopping rule for hierarchical cluster analysis, a cut-off point was determined according to the dendrogram to signify when the clustering process should be stopped [53]. Next, we used information from the agglomeration table and the dendrogram to determine the number of clusters. Results suggested three-cluster and four-cluster solutions, respectively. Next, the K-means cluster algorithm was applied to improve results from the hierarchical procedures to provide more accurate cluster membership. The K-means algorithm defined three groups using the initial seed points from the hierarchical clustering.

Table 2 shows the final cluster centers. For each cluster, the centroid (mean value) is provided. In absolute terms, clusters were dissimilar, ranging from 2.548 (cluster 1 vs. 2) to 3.452 (cluster 2 vs. 3) and to 4.648 (cluster 1 vs. 3). The greater the distance between the two clusters, the greater the differences in these clusters. The first cluster classifies N = 20 cases, the second N = 16, and the third N = 27. In detail, the first cluster rightly classifies AD patients (N = 17 of 20, or close to 85%). This cluster was characterized by low scores on neuropsychological measures, alpha and beta bands, and positive values of delta and theta bands. The second did not classify a specific diagnostic class of cases, with a mixed composition of patients with MCI, FTD, and AD. This cluster was characterized by low scores on neuropsychological measures, delta and theta bands, and positive values of alpha and beta bands. Finally, the third cluster classifies control cases (N = 15 of 19, or close to 79%) with good performance on neuropsychological measures, positive values on alpha and beta bands, and negative values on delta and theta bands. Results suggest that it is feasible to group cases based on patterns of cognitive performance and EEG bands.

#### 3.2.6. Clusters Description

After pooling cases for cluster membership, we ran analyses to explore the new classification. First, we investigated the neuropsychological and neurophysiological patterns of the three clusters by entering the neuropsychological scores and EEG bands in a series of ANOVAs with cluster membership as a factor (see Table 3). The three clusters differed for all neuropsychological measures, with effect sizes ranging from η^2^_p_ = 0.278 to 0.679. In detail, LSD post hoc comparisons revealed that the tree clusters significantly differ on all neuropsychological measures, except for short-term memory (cluster #1 vs. #2, *p* = 0.802), visual-spatial memory (cluster #3 vs. #2, *p* = 0.057), and semantic verbal fluency (cluster #1 vs. #2, *p* = 0.059). 

We found a slight effect of alpha (F_(2,60)_ = 4.014; *p* = 0.023; η^2^_p_ = 0.118) and delta bands (F_(2,60)_ = 5.276; *p* = 0.008; η^2^_p_ = 0.150) that did not survive to the post hoc procedures (all *p* > 0.05). The clusters differed for the remaining bands with a small effect size, η^2^_p_ = 0.301 (beta) and η^2^_p_ = 0.287 (theta). LSD post hoc comparisons on EEG bands revealed not-significant post hoc differences on the beta band between cluster #3 vs. #1 (*p* = 0.522) and on the theta band between cluster #3 vs. #2 (*p* = 0.676).

A non-parametric ANOVA was also used to test the strength of parametric analysis results, leading to the same conclusions. 

## 4. Discussion

The aims of the present study were twofold: firstly, to examine whether EEG patterns can be significantly different, allowing to discriminate between patients with AD, FTD, and MCI, compared with control subjects. Secondly, to examine, from a transdiagnostic perspective, the feasibility of clustering group cases based on patterns of cognitive performance and EEG bands independent of the diagnostic group.

Concerning the first aim, the results indicated that the theta band represents an EEG pattern allowing discrimination of the three clinical groups. Precisely, results showed lower theta activity in posterior areas and greater theta activation in the FTD group compared with the MCI group. The AD and FTD groups, which generally had similar activation, showed greater involvement of theta bands than the control subjects. The MCI group showed lower involvement of theta bands than the other groups, as did the control subjects. There were also some differences in the case of the delta band. AD patients showed more activity in temporal-occipital areas than controls and MCI patients. By contrast, the alpha and beta bands did not discriminate among the AD, FTD, and MCI groups. Overall, these results are consistent with previous findings, indicating increased power in lower frequency bands (delta and theta) in patients with AD [17,18,19]. Moreover, the results indicated that the differential activation of the frequency bands was related to the brain location in each group. Thus, the AD and control groups differed in temporal-occipital areas, with more activity in the AD group than in controls in C3, T5, T6, O1, and O2. The AD group showed higher delta activity than MCI patients in F8, C3, C4, T4, T5, P4, T6, O1, and O2.

Concerning the second aim, the cluster analysis indicated that it is feasible to group cases based on patterns of cognitive performance and EEG bands. In general, one may see cognitive decline as lying on a continuum that partially cuts across diagnostic categories but is also mixed with the wide inter-individual variability characteristic of aging. Thus, while patients with AD predominantly appeared in cluster #3 and controls mainly in cluster #1, there was substantial variability in both these clusters. Cluster #2 was characterized by an intermediate level of performance and featured a mixed composition of patients with MCI, FTD, and AD, as well as controls. As expected, several neuropsychological indicators were sensitive to detect the difference between clusters #3 and #2: short- and long-term memory, attention, semantic and phonemic verbal fluency, praxis, and fluid intelligence. Thus, neuropsychological tests prove quite sensitive in capturing an initial cognitive decline, although further worsening in performance occurred in several cognitive areas. Notably, beta and theta data also contributed to defining the composition of the clusters. Thus, an increase in theta band activity was uniquely associated with the difference between clusters #2 and #1. Therefore, it appears that neuropsychological tests are most sensitive in capturing an initial cognitive decline, while increased EEG theta activity is uniquely associated with a substantial worsening of the clinical picture and be considered a negative prognostic factor. Recent evidence is consistent with the idea that EEG biomarkers can be particularly suited to mark the progression of MCI and AD [15]. Overall, in line with the RDoC perspective, using both cognitive and neurophysiological data may provide converging evidence to document the decline of cognitive skills in at-risk individuals [17,18,19].

Altogether, the results of the present study are consistent with previous findings indicating that the qEEG analysis allows the identification of the indices of brain electrical activity changes related to pathological processes implicated by different types of dementia [20]. Moreover, this study indicated that the coupling between EEG and cognitive abilities might provide information on the cognitive alterations area that may be considered prodromal to dementia [54,55]. Notably, most previous studies either focused on EEG measures or neuropsychological analysis. We believe that by using tests with different methodological approaches (neuropsychological analysis and qEEG) we can better characterize the dysfunction of the brain system. On one hand, the use of qEEG is economical, fast, reproducible, and yields precise data in neurology and psychiatry that can be compared over time [56]. In line with previous observations [14,15,16], we observed that the QEEG data (delta and theta bands and their spatial distribution) allow a distinction between FTD, AD, and healthy aging controls. As expected, consistent group differences among groups were also observed using a comprehensive neuropsychological battery over and above differences in MMSE. Using multiple sources of information is one of the tenets of the recent RDoC approach [31] to classify mental disorders over and above classical diagnostic categories (as in the DSM-5 [5]). Indeed, in recent years, studies have begun to couple EEG measures with behavioral measures, for example, to test the attentional networks in subjects with attention-deficit/hyperactivity disorder [57]. In the dementia realm, a recent study based on the transdiagnostic perspective focused on the interrelations among neuroimaging data and clinical symptoms of apathy and disinhibition [58]. To analyze EEG and behavioral data from a unitary perspective, we referred to the clusters model approach [50]. While this approach found a large use in applied psychology (e.g., [51], to the best of our knowledge, it is the first time that the clusters model approach has been used to jointly examine neurophysiological and neuropsychological measures in dementia.

In the present study, the clusters model approach has enabled us to identify differentiated subgroups with specific neuropsychological patterns and EEG characteristics. This approach highlighted how diagnosis classification performed using routine measures may be less accurate and may not consider the complexity of the phenomena. Due to the small number of participants in this dataset, the inclusion of this classification approach enabled the flexibility and scalability of this method to accommodate several input features. This approach could integrate the MMSE criteria in a first diagnostic evaluation process and provide a step forward in classifying patients when more than one neuropsychological feature and EEG patterns are available. It is also important for future research to improve model parsimony and its interpretability. Overall, the use of this knowledge can increase the specificity of the first-level evaluation, allowing for a better selection of patients who need a subsequent evaluation using focused biomarkers.

The present study has a limitation related to the sample size. The power analysis indicated that the study had adequate statistical power at a large effect size level but low power at a small to moderate effect size. The small sample size indicates the need for caution in generalizing the findings of this study to the population with dementia, particularly considering the different types of disorders. Future studies with larger samples, possibly with multi-centric collaborations, are needed to obtain reliable results for the different groups of patients.

## 5. Conclusions

In conclusion, the present study indicates that EEG resting state activity, associated with different brain locations, can be a significant marker for discriminating patients with AD, FTD, and MCI against controls. In line with the RDoC perspective, considering jointly neuropsychological and EEG data may increase the possibility of documenting critical moments in cognitive decline, favoring the distinction between typical aging and dementia. We propose that this may be a promising line of investigation for future studies using multivariate classification methods [50] or machine learning algorithms [13], which allow to go beyond the classical diagnostic categories and to jointly evaluate both EEG resting state activity and neuropsychological performances.

## Figures and Tables

**Table 1:** Socio-demographic data and neuropsychological data of patients with AD, FTD, and MCI and the control participants. MMSE values indicate raw data; all other neuropsychological tests refer to equivalent scores. The means (M) and standard deviations (SD) are presented for all variables except sex (raw proportion). For each variable, the group effect and the effect size are reported. Analyses are one-way ANOVAs, except for gender (X^2^). In the case of significant group effects, LSD post hoc comparisons are reported as superscript letters: different letters indicate significantly different means.

	AD Group *N* = 19		FTD Group *N* = 7		MCI Group *N* = 18		Control Group *N* = 19		Group Effect	Size Effect
	M	SD	M	SD	M	SD	M	SD	*p*	η^2^_p_
Age	68.9	7.5	68.3	8.0	65.7	8.2	70.4	5.9	n.s.	0.06
Years of schooling	8.5	3.5	9.1	4.4	8.7	4.1	8.0	3.5	n.s.	0.01
Gender (M/F)	7/12		5/2		12/6		11/8		n.s.	
MMSE	19.2 ^c^	3.1	22.5 ^b^	2.2	26.6 ^a^	2.0	26.3 ^a^	1.4	<0.001	0.69
Long-term memory	0.6 ^b^	1.3	1.6 ^a^	1.4	2.1 ^a^	1.1	2.6 ^a^	1.1	<0.001	0.33
Short-term memory	1.1 ^b^	1.4	1.0 ^b^	1.5	1.3 ^b^	1.1	2.7 ^a^	1.0	<0.001	0.27
Visual-spatial memory	0.5 ^b^	0.7	1.1 ^a^	1.6	1.7 ^a^	1.1	2.0 ^a^	0.9	<0.001	0.26
Attention	0.4 ^b^	0.9	1.0 ^b^	1.7	2.2 ^a^	1.6	2.5 ^a^	1.1	<0.001	0.35
Semantic verbal fluency	0.5 ^b^	0.7	0.7 ^b^	0.7	1.9 ^a^	1.6	2.3 ^a^	1.0	<0.001	0.32
Phonemic verbal fluency	0.6 ^b^	1.2	0.9 ^b^	1.6	2.4 ^a^	1.3	2.4 ^a^	0.7	<0.001	0.36
Constructional praxis	0.1 ^c^	0.4	0.6 ^c^	0.8	1.9 ^b^	1.7	3.3 ^a^	1.2	<0.001	0.56
Fluid intelligence	1.0 ^b^	0.6	1.5 ^a,b^	1.0	2.1 ^a^	1.2	2.7 ^a^	1.3	<0.001	0.29

**Table 2:** Final composition of the cluster centers solution.

	Cluster		
	1	2	3
Long-term memory	−0.871	−0.049	0.674
Short-term memory	−0.610	−0.646	0.834
Visual-spatial memory	−0.784	0.177	0.476
Attention	−0.750	−0.620	0.923
Semantic verbal fluency	−0.644	−0.368	0.695
Phonemic verbal fluency	−0.802	−0.345	0.798
Constructional praxis	−0.790	−0.511	0.888
Fluid intelligence	−0.748	−0.526	0.866
EEG alpha bands	−0.677	0.563	0.168
EEG beta bands	−0.425	0.215	0.187
EEG delta bands	0.580	−0.572	−0.090
EEG theta bands	0.789	−0.301	−0.407
*N*	20	16	27

Note. Single EEG bands were saved from PCA factor scoring procedures.

**Table 3:** ANOVAs for cluster membership. In the case of group effects significant after Bonferroni correction, LSD post hoc comparisons are reported as superscript letters: different letters indicate significantly different means.

	Cluster	M	SD	Df	F	*p*	η^2^_p_
Long-term memory	1	−0.674 ^c^	0.769	2	23.883	<0.001 *	0.443
	2	−0.067 ^b^	0.893				
	3	0.842 ^a^	0.639				
Short-term memory	1	−0.548 ^b^	0.748	2	25.835	<0.001 *	0.463
	2	−0.395 ^b^	0.845				
	3	0.918 ^a^	0.667				
Visual-spatial memory	1	−0.575 ^b^	0.620	2	11.547	<0.001 *	0.278
	2	0.082 ^a^	0.944				
	3	0.623 ^a^	1.039				
Attention	1	−0.763 ^c^	0.541	2	47.581	<0.001 *	0.613
	2	−0.160 ^b^	0.793				
	3	1.011 ^a^	0.611				
Semantic verbal fluency	1	−0.658 ^b^	0.576	2	22.258	<0.001 *	0.426
	2	−0.075 ^b^	0.741				
	3	0.828 ^a^	0.967				
Phonemic verbal fluency	1	−0.824 ^c^	0.629	2	32.428	<0.001 *	0.519
	2	0.288 ^b^	0.958				
	3	0.777 ^a^	0.579				
Constructional praxis ^¶^	1	−0.795 ^c^	0.284	2	81.560	<0.001 *	0.699
	2	−0.222 ^b^	0.760				
	3	1.091 ^a^	0.637				
Fluid intelligence ^¶^	1	−0.702 ^c^	0.374	2	48.220	<0.001 *	0.679
	2	−0.403 ^b^	0.504				
	3	1.105 ^a^	0.782				
EEG alpha band	1	−0.402	0.789	2	4.014	0.023	0.118
	2	0.366	1.004				
	3	0.225	1.091				
EEG beta band ^¶^	1	−0.420 ^a^	0.671	2	6.956	<0.001 *	0.301
	2	0.951 ^b^	1.328				
	3	−0.151 ^a^	0.593				
EEG delta band	1	0.350	0.951	2	5.276	0.008	0.150
	2	−0.636	0.633				
	3	0.020	1.081				
EEG theta band	1	0.625 ^b^	0.996	2	12.092	<0.001 *	0.287
	2	−0.584 ^a^	0.494				
	3	−0.341 ^a^	0.872				

Note. ^¶^ Welch adjustment for unequal variance. * Bonferroni corrected *p* < 0.05.

## Data Availability

Data will be made available only upon request for reasons of privacy.
